# Supplementary material for: Integration of population-based surveys for neglected tropical diseases: A scoping review
Source: PLoS Negl Trop Dis. 2026 Apr 6;20(4):e0013733. doi: 10.1371/journal.pntd.0013733 (PMC13068340; doi:10.1371/journal.pntd.0013733)
Supplement: S1 Appendix — (PDF) [file pntd.0013733.s002.pdf]

S2 Appendix – Search strategy for PubMed

((integrat*[Title/Abstract]) AND (survey[Title/Abstract])) AND ("neglected tropical disease*"[Title/Abstract] OR NTD[Title/Abstract] OR "Buruli ulcer"[Title/Abstract] OR "Chagas"[Title/Abstract] OR "dengue"[Title/Abstract] OR "chikungunya"[Title/Abstract] OR "dracunculiasis"[Title/Abstract] OR "guinea-worm"[Title/Abstract] OR "echinococcosis"[Title/Abstract] OR "foodborne trematode infections"[Title/Abstract] OR "human African trypanosomiasis"[Title/Abstract] OR "sleeping sickness"[Title/Abstract] OR "leishmaniasis"[Title/Abstract] OR "leprosy"[Title/Abstract] OR "lymphatic filariasis"[Title/Abstract] OR "mycetoma"[Title/Abstract] OR "noma"[Title/Abstract] OR "onchocerciasis"[Title/Abstract] OR "river blindness"[Title/Abstract] OR "podoconiosis"[Title/Abstract] OR "non-filarial elephantiasis"[Title/Abstract] OR "rabies"[Title/Abstract] OR "scabies"[Title/Abstract] OR "other ectoparasitoses"[Title/Abstract] OR "schistosomiasis"[Title/Abstract] OR "snakebite envenoming"[Title/Abstract] OR "soil transmitted helminth"[Title/Abstract] OR "sporotrichosis"[Title/Abstract] OR "taeniasis"[Title/Abstract] OR "cysticercosis"[Title/Abstract] OR "trachoma"[Title/Abstract] OR "tungiasis"[Title/Abstract] OR "yaws"[Title/Abstract])
