# Supplementary material for: Integration of population-based surveys for neglected tropical diseases: A scoping review
Source: PLoS Negl Trop Dis. 2026 Apr 6;20(4):e0013733. doi: 10.1371/journal.pntd.0013733 (PMC13068340; doi:10.1371/journal.pntd.0013733)
Supplement: S2 Appendix — (PDF) [file pntd.0013733.s003.pdf]

S3 Appendix - Data extraction form

	Information	
1	Author(s)	
2	Year of publication	
3	Title	
4	Journal/publisher	
5	Types of evidence source	
6	Origin/country of origin	
7	Objective/ primary aim of the survey	
8	Context	
9	Participants	
10	Methods	
11	NTDs/diseases covered in the study	
12	Key findings that relate to strategies used to integrate multiple NTD surveys	
