# Supplementary material for: Integration of population-based surveys for neglected tropical diseases: A scoping review
Source: PLoS Negl Trop Dis. 2026 Apr 6;20(4):e0013733. doi: 10.1371/journal.pntd.0013733 (PMC13068340; doi:10.1371/journal.pntd.0013733)
Supplement: Attachment — Submitted filename: Response to reviewers.pdf [file pntd.0013733.s006.pdf]

Amanuel Belay
CDT-Africa
8th Floor, College of Health Sciences Building
Tikur Anbessa Hospital compound, Addis Ababa, Ethiopia
Phone: +251 966 253760; Email: amanbelays@gmail.com

24th February 2026

Dr Shaden Kamhawi
Editor-in-chief
PLOS Neglected Tropical Diseases

Dr Shaden Kamhawi

Re: Author response to reviewers' comments on manuscript:
'Integration of population-based surveys for Neglected Tropical Diseases: A Scoping Review'

Thank you for considering our manuscript for publication in PLOS Neglected Tropical Diseases. We are grateful to the reviewers for their time and expertise, and we believe that the revisions have strengthened the manuscript.

Please find our point-by-point responses to the comments below. In addition to the requested changes, we have re-organised the Discussion section (without making major changes to the content) so that the order of the paragraphs follows the order of our review questions. We believe this restructuring improves the overall readability and flow of the manuscript. We have uploaded both a clean version and a tracked-changes version of the revised manuscript to the Editorial Manager system.

We hope that the revised manuscript meets the standards for publication in your journal.

Yours sincerely,

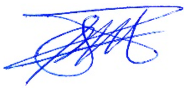

Amanuel Belay Simane

Point-by-point response

We sincerely thank the Editor and Reviewers for their careful review of our manuscript and for the insightful and constructive comments provided. We have carefully considered each comment and revised the manuscript accordingly. We believe that these revisions have strengthened the quality and clarity of the manuscript, and we greatly appreciate your valuable contributions.

Reviewers' Comments:

Methods

Reviewer #3: The inclusion criteria were not completely clear as laid out in the methods section. The authors state that they were targeting surveys with multiple NTDs. However, from a review of the reference section there were papers that were just one NTD along with malaria for example (reference 20).

Further, how did they deal with multiple publications from the same set of surveys? For example, I am familiar with the schistosomiasis literature and was able to identify two studies that should have been considered in this scoping review. The first was a survey done in one zone of Amhara, Ethiopia, and the second was a set of surveys done in another nine zones of Amhara, Ethiopia, to complete the map. It seems like the second paper was dropped. It is not clear why.

King JD, et al. Intestinal parasite prevalence in an area of ethiopia after implementing the SAFE strategy, enhanced outreach services, and health extension program. PLoS Negl Trop Dis. 2013 Jun 6;7(6):e2223. doi: 10.1371/journal.pntd.0002223. PMID: 23755308; PMCID: PMC3675016.

Nute AW, et al. Prevalence of soil-transmitted helminths and Schistosoma mansoni among a population-based sample of school-age children in Amhara region, Ethiopia. Parasit Vectors. 2018 Jul 24;11(1):431.

Response: *We agree that the inclusion criteria should have been more explicit and we have altered the way the inclusion criteria are described. Integrated surveys that included at least*

one NTD were also included in the study. We have revised the inclusion criteria accordingly from lines 124 - 125

Where multiple publications originated from the same underlying study, only the publication most relevant to the objectives of this scoping review was used for data extraction. Relevance was determined based on the extent to which the publication directly addressed the review questions and reported outcomes of interest. Companion papers reporting secondary analyses or findings not aligned with the review objectives were identified but not extracted to avoid duplication of evidence.

For example, from the following companion studies, we only included Sime et al because it discusses the lessons learned from the lymphatic filariasis and podoconiosis integrated study conducted in Ethiopia

Sime H, et al. Integrated mapping of lymphatic filariasis and podoconiosis: lessons learnt from Ethiopia. Parasit Vectors. 2014 Aug 27;7:397. doi: 10.1186/1756-3305-7-397. PMID: 25164687; PMCID: PMC4153915.

Deribe K, et al. Epidemiology and individual, household and geographical risk factors of podoconiosis in Ethiopia: results from the first nationwide mapping. Am J Trop Med Hyg. 2015 Jan;92(1):148-158. doi: 10.4269/ajtmh.14-0446. Epub 2014 Nov 17. PMID: 25404069; PMCID: PMC4288951.

We have included the following statement in the methodology section from lines 170 - 175.

"For companion studies, where multiple publications originated from the same single survey, only the publication most relevant to the review questions was selected for data extraction to avoid duplication of evidence. Relevance was determined based on the extent to which publications reported outcomes of interest. Companion papers reporting secondary analyses or findings not aligned with the review objectives were identified but not extracted to avoid duplication of evidence."

The reviewer is correct that the study by Nute et al (2018) meets the inclusion criteria. However, it was not identified by the database search because the term "integrat" was not included in the title, abstract, or MeSH terms. Although the study does consist of integrated*

surveys, the manuscript does not discuss any challengers or enablers of integration, which were key questions for our scoping review. These concepts are covered in more detail in the study by King et al (2013), which, as the reviewer explained, covers a subset of the surveys in the manuscript by Nute et al.

Results

Reviewer #3: The main addition that is needed for this manuscript is the table that describes the included studies and the relevant information that was extracted. This is commonly included in reviews such as this one. This should be a table of 81 rows and should report the information that was reviewed such as year of study, age range, type of assessments, NTDs, etc (i.e. the variables on the S3 data extraction form). This is listed as having been done in the PRISM document, but it is not present in this submission. The authors can include this as appendix or in the main text file, but it needs to be included so the reader knows which studies were detected. It is too difficult for the reader to parse through the results text and the reference section to piece together the nature of the studies included. Having this table available will be helpful to future researchers.

The authors rightly note that a key challenge to integrating surveys is that target age groups differ. It would be very helpful to the reader if the authors could include a table displaying the required or suggested age groups for the various surveys they reviewed. This would really help make the point about how survey design will be important to reach the required age groups.

Response: *We agree that this is important information and have included a table that reports the extracted information from the articles as a supplementary document. Data extraction was done on a separate sheet for the integrated prevalence surveys papers and the other articles (review, consultation summary articles, qualitative document review, and guideline). The summary table includes the target age groups for each integrated survey as well. We have also included in the main manuscript a table that shows the target age groups for the different survey types for selected NTDs from line 396.*

Conclusions

Reviewer #3: It was often difficult to tell the difference between the results section and the discussion section. There were instances in the results where the authors moved away from presenting results and moved more towards commenting on the results. For example, see line 245-249 where the authors compare their approach with past approaches, something that would normally be found in the Discussion. Whereas in the Discussion lines 553-558, the authors introduce mHealth for the first time. This reads more like a finding or result of the scoping review and should be moved up into the results section.

Response: *We thank the reviewer for the suggestion to more clearly separate the content of the Results and Discussion section. We have removed the sentence reflecting on the current study from the results section (from 217), but have retained the part presenting a simple description of the characteristics of the five review papers included within the scoping review.*

Regarding digital health technologies, while no sources in the scoping review explicitly reported the use of mHealth for integrated NTD surveillance. As such, references to mHealth are retained in the Discussion section (lines 538–534). We have revised the discussion section to make it more of a recommendation rather than a finding from the scoping review.

The authors cover the MBA for serology in the results section. It would have been helpful to discuss the potential limitations of this approach in the discussion. Potentially in the paragraph around limited funding for NTD programs. These machines are expensive to keep supplied and to keep functioning. Second there are the decisions that go into which antigens to place on the beads, and those decisions are likely tied to where funding is coming from. Lastly, some of the antigens are still not completely agreed upon (see LF, Dengue). Some of these concerns should be raised so that there is a balanced assessment of the MDA approach.

Response: *We have included the potential challenges of MBA in our discussion section from line 544 – 549.*

The authors focus most of their manuscript on the PC-NTDs. This is likely justified given that most of the papers they found (per Fig 3) were among those diseases. However, it would be helpful to have their thoughts on how to integrate monitoring and evaluation or surveys for

the other diseases. Is it that integration is not as important for those diseases, or is it that no one has tried? More discussion on this would be enlightening.

Response: *We thank the reviewer for the important suggestion. We agree that our study identified more evidence on the integration of PC-NTDs. This might be due to the need for population-based surveys for the identification of areas for MDA. We have included this in the discussion section and discussed how other NTDs surveys can be integrated from lines 508 - 520*

Editorial and Data Presentation Modifications?

Reviewer #1: Thank you for the opportunity to review this interesting paper. I have been trying to push for survey integration for many years. It is extremely difficult, as the authors note. I think this publication can help.

The authors should be congratulated for completing a big review and for the even analysis and nice presentation. My comments are minor.

39-40 : The Global Trachoma Mapping Project was a specific, time-limited project to map the baseline prevalence of signs of trachoma in populations in which this had not previously been done, funded by DFID and USAID. It finished in 2016. Suggest replace "and the Global Trachoma Mapping Project" with "and standardized trachoma prevalence surveys". 54: suggest change the word "regions" (which has political connotations in some countries), with "areas" or similar.

Response: *Thank you for your comments. We have replaced "and the Global Trachoma Mapping Project" with "and standardized trachoma prevalence surveys".*

81-82: extremely pedantic point from me here. Suggest list the diseases either in alphabetical order or the order in which the donations were made (since this is a sentence about history) – if you choose the latter, oncho would come first, but I am not sure about the order of the others.

Response: *We have listed the diseases in alphabetical order.*

83-84: “Population-based prevalence surveys are … also used to monitor and evaluate the impact of control programs”. We use that language (about surveys evaluating impact) all the time, but in fact this is not really the role of repeated surveys, which is, in my view, always forward-facing – it’s to allow programmes to make decisions on what to do next, to stop or continue interventions. Evaluation of impact can only be done in a setting in which the simultaneous effect of other exposures can be controlled for.

Response: *We fully agree that the primary role of surveys is to guide forward action, and have stated this point more clearly. We do also feel that surveys can play a role in evaluating the impact of control, though we acknowledge the difficulties in controlling for other exposures. We have rephrased the two sentences:*

“The primary role of population-based prevalence surveys for PC-NTDs is to identify populations in need of MDA and to guide decisions about the required frequency of PC. They are also used to generate insights on disease epidemiology and burden and the impact of control programs”

87: This point will seem pedantic, but to me is extremely important. If we say, “Surveys drive up the cost of control and elimination programmes…” it suggests that surveys are somehow *outside the programme* and therefore optional extras or luxuries, perhaps done to satisfy the whims of academics or interested bystanders. In fact they are an essential part of the programme; MDA without surveys would be reckless, because the likely future benefit of the intervention would be unknown and the costs (money, time, adverse events, AMR) are non-zero. You have made the case for surveys in the previous para. Please don’t sweep that away here.

Response: *We fully agree that surveys are an integral component of MDA programmes and have rephrased the sentence so that it does not read as though we are positioning them as an add-on. Nonetheless, we feel that it is essential to make the point about the high costs of surveys within this paragraph.*

“While being essential to ensure that resources for control are well-targeted, surveys constitute a large proportion of the costs of control and elimination programmes.”

220: “two were a perspective article” – suggest edit to “two were perspective articles”

Response: *We have revised it to "two were perspective articles"*

282: suggest change "collected" to "collecting"

Response: *We have revised it to "collecting"*

289: I appreciate that this is a particular interest of the research group, but strictly speaking, podoconiosis is not an NTD in its own right, as least, not an NTD on the WHO list

Response: *We have explained why we included podoconiosis within this review in the inclusion criteria (lines 125-127).*

295-298: "To support this, WHO has developed "Recognising neglected tropical diseases through changes on the skin', a simple tool focusing on major skin changes such as ulcers, lumps, swollen limbs, and patches, and provides a diagnostic flowchart and algorithm for detecting and managing NTDs, can be used for mapping activities". This sentence needs some editing to make complete sense (though the meaning is clear.) It might be easier to break it into shorter sentences.

Response: *We have revised it to "To support this, WHO has developed "Recognising neglected tropical diseases through changes on the skin', a simple tool focusing on major skin changes such as ulcers, lumps, swollen limbs, and patches. The tool provides a diagnostic flowchart and algorithm for detecting and managing NTDs and can be used for mapping activities"*

298-299: "which was developed by WHO" is redundant here, the point having been noted in the previous sentence.

Response: *We have removed "which was developed by WHO" and revised the sentence to "This tool is intended to be used by frontline non-specialist health workers with minimal dermatological training."*

308-318: the Tropical Data collaboration, which took over trachoma prevalence survey support when the Global Trachoma Mapping Project finished and supports health ministries around the world to conduct several hundred district-level trachoma prevalence surveys each year, is potentially one of the most useful platforms for integration – because the trachoma

survey process is rigorously quality controlled and quality assured, and now well-grooved. It would be worth mentioning here.

Response: *We have included "trachoma mapping survey" as an important existing platform for the integration of NTDs survey. We have included the following from lines 378-381.*

"Currently, trachoma mapping is supported by Tropical Data, a consortium of partners that supports countries' Ministries of Health to conduct epidemiologically robust, high-quality, WHO-standardised prevalence surveys."

353: same comment as for 39-40 – the GTMP was funded, but is no longer "existing". Please also see the comment above about Tropical Data, which may be worth referring to here instead of the GTMP

Response: *We have replaced GTMP with "standardised trachoma prevalence surveys".*

394: ITM stands for integrated threshold mapping

Response*: We have corrected it to Integrated Threshold Mapping (ITM).*

403: I think the major challenge to integration is lack of will to make it happen, but that's a personal view that is probably not identifiable in the literature that you identified

Response: *We have addressed this under "Programmatic and organisational barriers" as follows: "Furthermore, there may be resistance to integration due to fear of loss of control, power or funding. In addition, since integration is still a relatively new concept in many programs, hesitancy from field team members to work together can also be another challenge." (lines 435 – 438).*

418: please delete the word "infection" after "trachoma"

Response: *We have deleted the word "infection"*

419: please change "measure" to "estimate" and delete "(follicular)"

Response: *We have changed this sentence as suggested*

420: please change “trachoma trichiasis” to “trachomatous trichiasis”

Response: *We have changed it to “trachomatous trichiasis”*

421: please change “schistosome infection” to “schistosomiasis”

Response: *We have changed “schistosome infection” to “schistosomiasis”*

423: please change “for” to “in”

Response: *We have changed it to “in”*

437-438: also, kids going to school may be systematically different in terms of risk than non-school-going kids

Response: *We agree that children attending school may differ systematically in terms of risk compared with those not attending school. However, we did not include this point in the results section because none of the included sources of evidence included in the review reported this as a challenge or source of bias.*

547 and 549: CSW – should this be CHW?

Response: *We have changed it to “CHW”*

568: * Recent global geopolitical shifts, such as reductions” – suggest change “such as” to “causing”

Response: *We have changed “such as” to “causing”*

586-587: “generating some information is much better than generating no information”. Statements similar to this are often used to justify collecting bad data. I disagree with it *as a general statement*. When decisions for populations are made on the basis of data collected (and they are), we need to collectively make sure that the data address questions that are programmatically relevant, and are interpreted in the right way. (The example that you subsequently give in this para about LF seems sound, however.)

Response: *We agree that this point may be interpreted in this way, which we did not intend. We have edited this to*

"In this new landscape of scarcity, NTD programs, donors and WHO focal points should encourage optimising resources, which may necessitate compromise on certain disease-specific survey protocols"

594: when abbreviated, WHO does not take the definite article. (The band does.) In other words, please delete the "the" in front of "WHO".

Response: *We have removed the word "the" before "WHO"*

Reviewer #3: Abstract, line 39-the platform for trachoma is officially called "Tropical Data." Suggest using this term throughout when referring to the ongoing trachoma surveys. GTMP was the original phase of baseline surveys, however, that project is now completed.

Response: *We have replaced GTMP with "standardised trachoma prevalence surveys".*

Introduction line 88- consider the use of the term "survey teams" instead of "health teams."

Response: *We have changed "health teams" to "survey teams"*

Line 87, while surveys do certainly cost money, in the end they can be cost saving as they can inform a program to stop costly MDA and other interventions, right?

Response: *We agree that surveys can ultimately be cost-saving by informing programmes when to scale down or stop costly MDA and other interventions. However, we think the crucial point is that they ensure resources are well-targeted, and have added this to the sentence from lines 88 -89:*

"While being essential to ensure that resources for control are well-targeted, surveys constitute a large proportion of the costs of control and elimination programmes."

Methods lines 119-121 are confusing. The authors state that they present findings of the second research question only, however, they clearly cover the other 3 points throughout the manuscript, as they have figures showing which countries the work has been done in, and sections discussing challenges. Can the authors please clarify what is meant, or remove the

sentence?

Response: *We agree that this part was confusing and have removed the sentence indicating that we focus on one research question within the OSF protocol.*

Lines 350-353 are very similar to lines 314-318 and make the same point with very similar wording. Please choose one location to make the point about the importance of having existing platforms.

Response: *We have removed the first paragraphs and revised the second paragraph (lines 315-324).*

Figure 2: The authors use the word publications in the title and the axes. Were the only papers identified those that were published? The search strategy included grey literature, no? If no grey literature articles were found, this should be reported.

Response: *We have revised the term "publication" to the more umbrella term "sources of evidence," in line with JBI and PRISMA-ScR guidance. Accordingly, "Number of publications per year" has been revised to "Number of sources of evidence included per year," and "number of publications" has been revised to "number of sources of evidence."*

Summary and General Comments

Reviewer #2: In this manuscript, Belay and colleagues present the results of a scoping review on integrated surveys for NTDs. Their review is thorough and the article is well written and includes a useful discussion of the barriers to integrating surveys. The authors also discuss the limitations of their review (e.g., the restriction to English-language articles).

My concerns about the paper are based on the dramatic shifts in the funding landscape that occurred in 2025. In the current context, it will be essential for the NTD community to radically change the way that M&E is done. In the face of funding shortfalls, disease-specific surveys will be very hard to justify – is there a donor that would pay $1M for an LF survey in one country at this point (e.g., line 390)? Integration of NTD surveys with malaria, VPD, and other surveys will be essential. These issues are noted in the text in a couple of places (e.g., a

paragraph on pages 22-23), but these mentions feel like an afterthought. I think this paper would be much more useful to readers if integrated surveys – beyond just NTDs - received more attention as a necessity in the current context.

Response: *Thank you for this comment. We agree and have added an explicit mention of this in the discussion (from line 573 "The identification of opportunities for integration should also be considered within broader health programmes seeking to optimise the use of scarce resources"). We do not discuss this in further detail as the main focus of this paper was on the integration of NTD surveys, and our findings are somewhat limited to this specific area.*
